# Capuchin and rhesus monkeys show sunk cost effects in a psychomotor task

**DOI:** 10.1038/s41598-020-77301-w

**Published:** 2020-11-23

**Authors:** Julia Watzek, Sarah F. Brosnan

**Affiliations:** 1 Department of Psychology, Language Research Center, Georgia State University, Atlanta, GA USA; 2 Department of Philosophy, Neuroscience Institute, Center for Behavioral Neuroscience, Georgia State University, Atlanta, GA USA

**Keywords:** Evolution, Psychology, Cognitive neuroscience, Decision

## Abstract

Human decision-making is often swayed by irrecoverable investments even though it should only be based on future—and not past—costs and benefits. Although this sunk cost effect is widely documented and can lead to devastating losses, the underlying psychological mechanisms are unclear. To tease apart possible explanations through a comparative approach, we assessed capuchin and rhesus monkeys’ susceptibility to sunk costs in a psychomotor task. Monkeys needed to track a moving target with a joystick-controlled cursor for variable durations. They could stop at any time, ending the trial without reward. To minimize the work required for a reward, monkeys should have always persisted for at least 1 s, but should have abandoned the trial if that did not yield a reward. Capuchin monkeys and especially rhesus macaques persisted to trial completion even when it was suboptimal, and were more likely to complete the trial the longer they had already tracked the target. These effects were less pronounced, although still present, when the change in expected tracking duration was signalled visually. These results show that sunk cost effects can arise in the absence of human-unique factors and may emerge, in part, because persisting can resolve uncertainty.

## Introduction

We routinely consider sunk costs (irrecoverable prior investments of e.g., money, time, or effort) when making decisions that should only be based on the future costs and benefits. For example, if your benefit from selling your car is bigger than from keeping it (e.g., due to lack of use or maintenance and repair costs), then you should sell it, regardless of what you initially paid for it. However, humans tend to persist in an endeavour the more resources we have already invested in it^1,2^. This susceptibility to sunk costs can lead to bad decision-making for individuals, organizations, and even societies as a whole, for example, if people spend time, money, and effort on doomed projects or policy initiatives. In one notable example, the Concorde airplane project wasted millions in funding even after decision-makers realised that it had become a “commercial disaster^3^;” in fact, this bias is sometimes called the Concorde fallacy. Here we assess capuchin monkeys’ and rhesus macaques’ susceptibility to sunk costs to better understand the mechanisms that underlie this phenomenon.

Such a comparative approach is particularly useful in this case because there are several psychological explanations for why this effect may arise that we can discriminate among by examining other species’ choice patterns. For example, people may justify continued investments because they have publicly committed to doing so, because they rationalize their previous decisions as sound rather than mistaken, because they want to avoid being wasteful, because they are uncertain about their prospects, or because they eschew a definite loss (if they sell lower than they bought) in case a small additional investment turns things around for a gain^1,2,4–13^. Disentangling which processes contribute to sunk cost effects empirically is difficult because multiple may play a role and because they make similar predictions—that people consider sunk costs when it is suboptimal to do so and that the effect increases with the size of the sunk cost (i.e., irrecoverable prior investment).

Assessing susceptibility to sunk costs in species other than humans can help us narrow down the possible explanations because other animals differ in some of these psychological mechanisms. If the sunk cost effect relies on human-unique factors, such as self-rationalization or public commitment, we would not predict other animals to show the sunk cost effect. On the other hand, if widespread responses to uncertainty or resource scarcity underlie the sunk cost effect, we would expect other animals to also be susceptible to sunk costs. We can further disentangle possible explanations by systematically studying the effect in species that vary in the behaviour of interest. For example, only starlings, capuchin monkeys, and rhesus macaques have shown human-like loss aversion under risk^14–16^, becoming more risk prone when options were framed as losses than when the same options were framed as gains. To the extent that loss aversion contributes to the sunk cost effect, we would expect it to emerge in these species, but not in others that do not respond to losses in this way (although this has not been widely studied in animals). Conversely, if susceptibility to sunk costs does not covary with differences in how a proposed mechanism is expressed across species, this suggests that this mechanism does not contribute to the emergence of the sunk cost effect in these species or in humans. Such a comparative approach can be particularly insightful if several mechanisms may contribute to the sunk cost effect in humans, because understanding the pattern of responses lets us assess their relative contributions.

Indeed, humans are not the only species that shows sunk costs effects^17^, suggesting that human-unique factors such as human language, culture, or formal economic markets are not required for this bias to arise. For example, in experiments in which pigeons and rats needed to complete a repetitive action (such as pecks or lever presses), both species showed sunk cost effects, persisting with the action even when it became optimal to abandon the reinforcement schedule by selecting an opt-out option that skipped to the next trial^8,18–21^ (but see Ref.^22^). However, this effect disappeared when uncertainty about the remaining investment (required pecks or lever presses) was removed by signalling, via colour change, that a specific number of actions had been completed^8,18^ or when persisting required many more responses to reward^8,18,19,23^. These results suggest that the sunk cost effect emerges, in part, when we are uncertain about when it becomes beneficial to opt out rather than to continue investing, especially if there is little cost to persisting. In a foraging task that required waiting (i.e., inaction rather than action), rats, mice, and humans also showed sunk costs effects, becoming more likely to complete a trial (i.e., to continue waiting rather than to opt out) the longer they had already waited^24^. Here, however, information about the remaining investment was always signalled via sound, suggesting that uncertainty reduction does not explain susceptibility to sunk costs in all contexts.

In this study, we tested capuchin and rhesus monkeys using a computer task to assess the sunk cost effect in nonhuman primates for the first time. These species make economically suboptimal choices similar to humans’ in some situations^25,26^ (e.g., framing effects^27^ and loss aversion^14^, endowment effects^28^, peak-end effects^29,30^ [but see Ref.^31^], sensitivity to counterfactual outcomes^32–34^). In other contexts, however, capuchin and rhesus monkeys are more likely than humans to abandon a learned strategy in favour of a more efficient one (e.g., switching to an optional shortcut^35^ or violating transitivity when it is optimal to do so^36^), suggesting that they may not be as susceptible to sunk costs as people are. Studying these two species therefore provides a unique opportunity to understanding the mechanisms that underlie the effect. Our psychomotor task required continued action to persist (similar, in some aspects, to the repetitive-action paradigms used with pigeons and rats^8,18,19,22^), but unlike previous work, it required a continuous action (maintaining pressure on a joystick to keep a cursor moving) rather than a discrete response (pecking or lever pressing). We implemented this change to encourage monkeys to perceive and assess the investment in its entirety and not potentially as a series of seemingly unconnected actions, only some of which were rewarded.

If monkeys are susceptible to sunk costs, we predicted that they would persist in tracking a target even when opting out was optimal, and that they would be more likely to persist the longer they had already persisted. Further, we explicitly tested the extent to which the sunk cost effect in capuchins and macaques may arise due to uncertainty about the required additional effort by signalling effort visually. Based on previous work in pigeons and rats, we expected monkeys to show smaller sunk cost effects in the signalled condition, when this uncertainty is removed, than in the unsignalled condition, when there is uncertainty.

## Methods

### Participants

We tested 26 capuchin monkeys (18 female, 8 male, age: *M* ± *SD* = 17.65 ± 8.06, range: 7 to ca. 45 years) and 7 rhesus macaques (all male, age: *M* ± *SD* = 23.57 ± 7.35, range: 16–37 years) at the Language Research Center of Georgia State University.

Capuchins were socially housed in mixed-sex groups, each with their own indoor/outdoor enclosures with a variety of climbing structures, visual barriers, and regularly provided enrichment devices (e.g., foraging boards and puzzle boxes). Capuchins had been trained to separate voluntarily into testing boxes attached to their indoor enclosures for cognitive and behavioural studies. They were never required to come into the test boxes for testing and they could choose not to participate at any time without consequences. Rhesus macaques were individually housed with continuous auditory and visual access to other monkeys and, when possible, regular social periods with compatible partners. Their enclosures doubled as testing boxes, but they too could choose not to participate at any time without consequences.

All monkeys had access to water at all times, including during testing, and were never food deprived (except for medical reasons unrelated to research studies). All testing food was given in addition to their daily diet of vegetables, fruit, and primate chow.

### Task

Monkeys were tested on individual computer testing systems following the procedures from our previous work^35–37^ (Supplementary Fig. S1; for details, see Ref.^38^). In this study, they needed to track a moving target with a cursor that they controlled with a joystick. After maintaining contact with the target for a specified duration, monkeys received a reward. If monkeys lost contact with the target, they did not receive a reward and the next trial began immediately.

**Figure 1:**
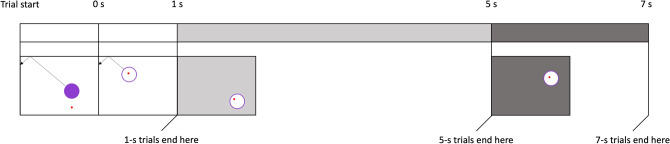
Schematic trial progression. Dotted line and arrow show an example trajectory of the target (not present on actual trials). The tracking duration started when the cursor entered the target and lasted 1, 5, or 7 s. If the target was tracked for the entire duration, the monkey was rewarded; if they lost contact with the target, the trial ended without reward. In the unsignalled condition, the background colour remained white throughout the trial. In the signalled condition (shown here), the background colour changed to light grey after 1 s and to dark grey after 5 s. For an animated example, see Supplementary Fig. S1.

All trials began with the target, a purple circle with a 150-pixel diameter, placed at a random location on an 800 × 600-pixel screen and a red cursor placed directly under the target at a distance of 85 pixels (Fig. 1 and Supplementary Fig. S1). While the cursor was outside the target, the target only moved when the cursor moved and stopped moving when the cursor stopped moving. The target moved in a straight line, starting with a random direction. When it reached the edge of the screen at a given angle, it reflected at the same angle, like a billiard ball bounces off a rail.

When the cursor entered the target, the target changed colour to white and now kept moving even when the cursor stopped. If the cursor lost contact, the trial ended without reward. If monkeys maintained the cursor within the target for a specified duration, they received positive auditory feedback (*whoop*) and a banana-flavoured food pellet. The next trial began immediately. Monkeys completed blocks of 24 trials.

During *training*, monkeys learned to track the target for variable durations, including but not limited to the tracking durations used during testing. The tracking duration required to receive a reward was a randomly determined number between 0 and X seconds for each trial (drawn from a uniform distribution and rounded to milliseconds, i.e., three decimal places). The maximum tracking duration possible (X) increased as monkeys proceeded through training; monkeys automatically proceeded to next phase when they successfully earned the reward in at least 80% of trials in two separate trial blocks (Table 1). Rhesus monkeys met the training requirement consistently sooner than capuchin monkeys, Cox proportional hazards: *b* ± *SE* = 1.84 ± 0.55, *OR *6.3, *p* < 0.001.

**Table 1:** Training phases.

Phase	Maximum duration	Species	Blocks to meet criterion			*N*_passed_	*N*_failed_
			*Mdn*	*IQR*	Range		
1	1 s	Capuchin	3	2–7	2–35	24	2
2	3 s	Capuchin	20	10.5–105.5	3–204	20	4
3	6 s	Capuchin	71	35–219.25	3–292	16	4
4	8 s	Capuchin	21	15–51	2–100	13	3
1	1 s	Rhesus	2	2–2	2–19	7	0
2	3 s	Rhesus	2	2–2.75	2–11	6	1
3	6 s	Rhesus	2.5	2–8.25	2–36	6	0
4	8 s	Rhesus	5	2.25–11.5	2–20	6	0

Maximum tracking duration required and monkeys’ training performance (number of blocks required to meet criterion and number of monkeys who did and did not meet the criterion) for each phase.

*Mdn *Median,* IQR *Interquartile range.

During *testing*, each block consisted of 12 baseline trials and 12 probe trials for all species. Baseline trials had a tracking duration of one second; probe trials had a tracking duration of either five (6 trials) or seven seconds (6 trials).

At the beginning of each trial, the expected value for the tracking duration was 3.5 s (1/2 chance of 1 s + 1/4 chance of 5 s + 1/4 chance of 7 s). The measure of interest was monkeys’ behaviour in probe trials after they had tracked the target for one second. At this point, they could have realized that it was not a baseline trial and that the expected additional tracking duration had changed (Table 2). Monkeys would now need to track the target another four or six seconds to receive the reward. But if they stopped moving the cursor to let the target lose contact (“opting out”), a new trial would begin that required only one second of tracking in most trials (and 3.5 s on average). Thus, opting out was optimal if there had not been a reward after one second of tracking. However, if monkeys persisted and tracked the target for a total of five seconds, they either received the reward at that time (in a five-second trial) or should have persisted for another two seconds (in a seven-second trial), which was less additional tracking time than a new trial required on average (3.5 s).

**Table 2:** Expectations for tracking durations.

Duration spent tracking target	Expected additional duration required	Calculation
0 s	3.5 s	$\frac{1}{2}\times 1\mathrm{s}+\frac{1}{4}\times 5\mathrm{s}+\frac{1}{4}\times 7s$
1 s	5 s	$\frac{1}{2}\times 4\mathrm{s}+\frac{1}{2}\times 6s$
5 s	2 s	$2s$

Expected values for additional tracking required to receive the reward depended on the duration already spent tracking and the likelihood for which trial duration was in effect.

In the *unsignalled* condition, the background colour remained white throughout the trial. In the *signalled* condition, the background colour changed when the expected duration changed (Fig. 1). In five- and seven-second trials, the background colour changed to light grey after the target had been tracked for one second. In seven-second trials, the background changed to dark grey after the target had been tracked for five seconds.

### Design

We used a within-groups design; each monkey completed both the unsignalled and the signalled condition. Half of the monkeys were assigned to complete the unsignalled condition first and half were assigned to complete the signalled condition first. Monkeys who passed all training phases completed 40 blocks per condition, resulting in a total of 1920 trials (960 × 1 s, 480 × 5 s, and 480 × 7 s). Monkeys could complete as many trials per session as they wanted and completed testing over several test days (capuchin *Mdn* = 6 days, *IQR* = 3–9 days, range = 2–11 days; rhesus *Mdn* = 2.5 days, *IQR* = 2–3 days, range = 1–3 days).

### Data analysis

To assess monkeys’ susceptibility to sunk costs, we fit a mixed effects logistic regression model (Model 1) with trial completion as the binomial outcome variable (did or did not track the target for the entire duration; i.e., did or did not earn the reward). We included individual identity as a random effect to account for individual variability in persistence. We included required tracking duration (1, 5, and 7 s), condition (unsignalled and signalled), species (capuchin and rhesus), block bin (each bin comprised 4 blocks, i.e., 96 trials; thus, monkeys’ first condition comprised bins 1–10 and the second comprised bins 11–20, centred to *M* = 0), and training duration (total number of blocks required to pass criterion, standardized to *M* = 0 and *SD* = 1) as fixed effects. We further included trial duration × condition × species and trial duration × condition × block bin interaction terms. We computed pairwise contrasts for significant model terms. We used likelihood ratio tests using single-term deletions to assess each factor’s importance with respect to model fit.

To assess monkeys’ susceptibility to sunk costs depending on how long they had already tracked the target, we first organized the data into seven non-exclusive subsets for sunk costs of 0, 1, 2, 3, 4, 5, and 6 s. That is, each subset contained data for trials at the points when monkeys had already tracked the target for at least 1–6 s, respectively. For example, sunk cost 0 s applied to all trials, and sunk cost 6 s applied to all trials for which monkeys had already tracked the target for at least 6 s (by definition, this subset could not include trials with tracking durations of 1 or 5 s). We then calculated the time remaining for the trial by subtracting the sunk cost from the required tracking duration. For example, if a monkey had already tracked the target for 3 s in a 5-s trial, the time remaining was 2 s. However, for a monkey to have 2 s remaining in a 7-s trial, they would have already tracked the target for 5 s. That is, 7-s trials had higher sunk costs than 5-s trials when the same amount of time was remaining. We excluded 1-s trials for this analysis because sunk costs were always at least 0 but never more than 1 s (i.e., there was no variability in sunk costs for these trials).

For the combined data, we then fit a mixed effects logistic regression model (Model 2) with trial completion as the binomial outcome variable (did or did not track the target for the entire duration; i.e., did or did not earn the reward). We included the trial duration × time remaining interaction as a fixed effect. We again included individual identity as a random effect to account for individual variability in baseline persistence and included the trial duration (5 and 7 s) × condition (unsignalled and signalled) × species (capuchin and rhesus) interaction as a covariate. We used a likelihood ratio test using single-term deletion to assess the importance of the trial duration × time remaining interaction with respect to model fit. We computed a pairwise contrast to compare the regression coefficients for 5- and 7-s trials. If monkeys were more likely to finish tracking the target the longer they had already tracked it, then the probability to complete the trial should be higher for higher sunk costs (i.e., higher in 7- than 5-s trials if the same time was remaining), and it should be less affected by the time still remaining. That is, the slope for 7-s trials should be shallower than for 5-s trials.

### Ethics

This study was purely behavioural, non-invasive, and was carried out in accordance with all applicable international, national, and institutional ethical guidelines and legal requirements. All procedures were approved by the Georgia State University Institutional Animal Care and Use Committee (IACUC A19028 and A20018 for capuchins and A19029 for rhesus). Georgia State University is fully accredited by the Association for Assessment and Accreditation of Laboratory Animal Care (AALAC).

## Results

### Effects of trial duration and signalling condition

We found a significant trial duration × condition × species interaction effect on monkeys’ likelihood to track the target for the required duration, χ^2^(2) = 17.22, *p* < 0.001, suggesting that species differed in whether they completed trials depending on how long it took to do so and whether this duration was signalled or not. We dropped the trial duration × condition × block bin interaction term and fixed effect of training duration, as they did not significantly improve model fit, full vs. reduced Model 1: χ^2^(3) = 3.87, *p* = 0.276. However, we found significant two-way interaction effects of block bin with trial duration, χ^2^(2) = 103.07, *p* < 0.001, and condition, χ^2^(1) = 15.14, *p* < 0.001, indicating that monkeys’ responses changed over time (see also Supplementary Tables S1 and S2).

**Figure 2:**
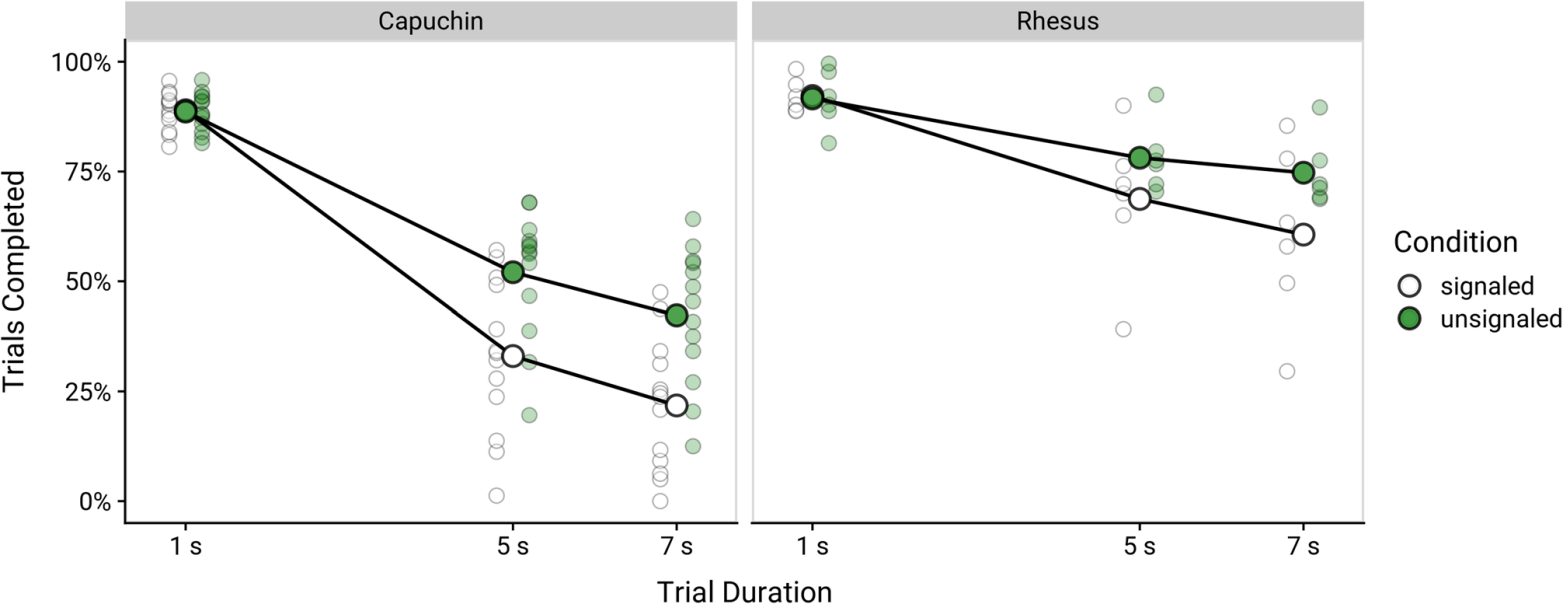
Trial completion by species, trial duration, and condition. Small transparent points indicate individual monkeys; large points connected by lines indicate group means.

Overall, monkeys completed almost all 1-s trials but were 7 times less likely to complete 5-s trials and 10 times less likely to complete 7-s trials (Fig. 2), odds ratios (*OR* ± *SE*) 5 s: 6.88 ± 0.26, 7 s: 10.25 ± 0.39, *p*s < 0.001. However, the magnitude of this effect depended on the species and the condition (for detailed results for all pairwise contrasts, see Supplementary Table S3). Although capuchin monkeys and rhesus macaques completed 1-s trials at similar, high rates, capuchins became 11 and 19 times less likely to complete 5- and 7-s trials, respectively (*OR* ± *SE* 5 s: 11.45 ± 0.45, 7 s: 19.04 ± 0.78, *p*s < 0.001). Rhesus macaques became only 4 times and 5.5 times less likely to do so (*OR* ± *SE* 5 s: 4.13 ± 0.27, 7 s: 5.52 ± 0.36, *p*s < 0.001), suggesting that they suffered more from sunk cost effects than capuchins. Further, when the trial duration was signalled through a change in background colour, rhesus macaques (*OR* ± *SE* 5 s: 1/0.79 = 1.27 ± 0.07, 7 s: 1.50 ± 0.06, *p*s < 0.001) and especially capuchin monkeys (*OR* ± *SE* 5 s: 2.37 ± 0.02, 7 s: 2.88 ± 0.02, *p*s < 0.001) became even more likely to opt out of the trial.

**Figure 3:**
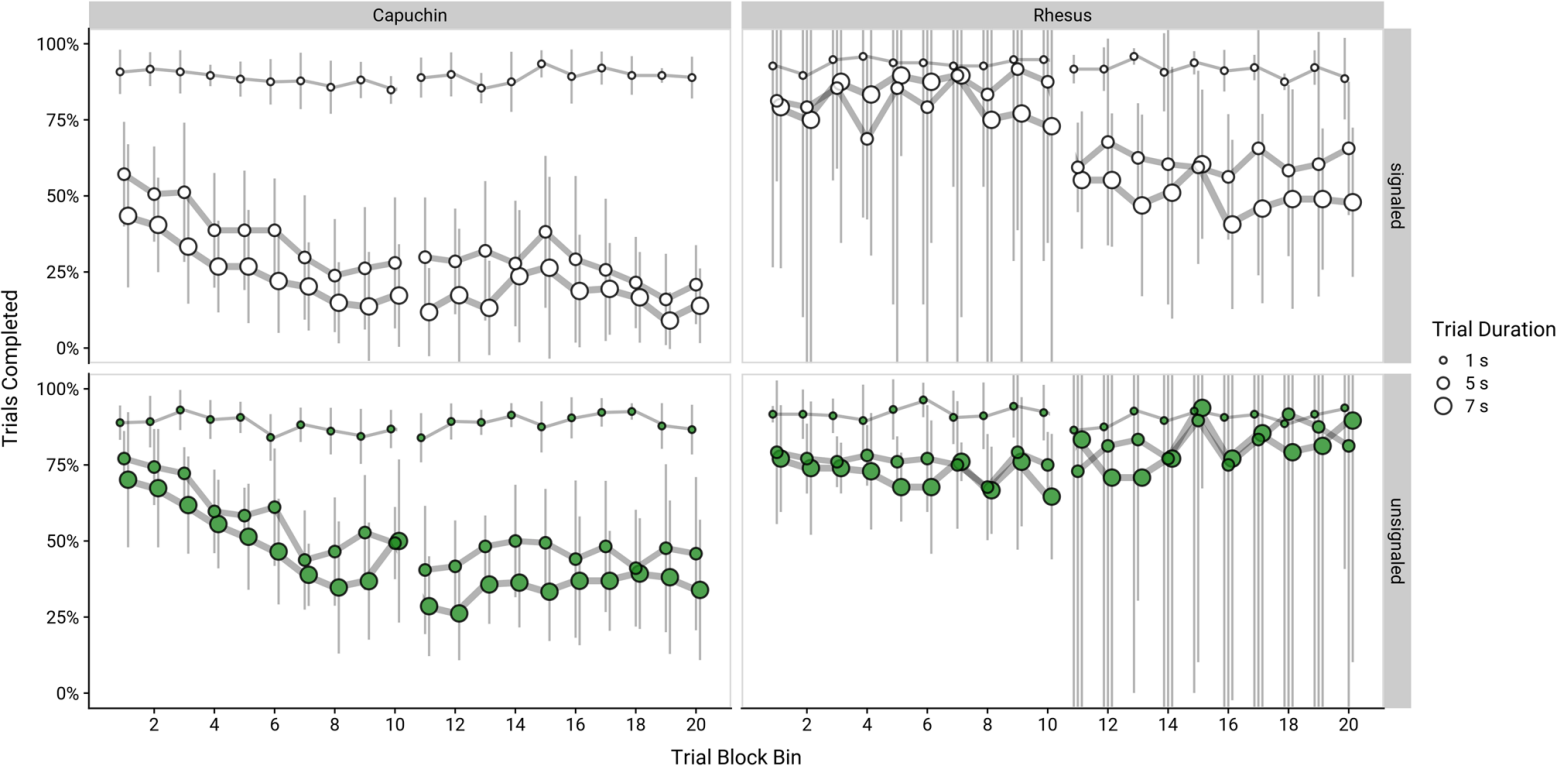
Trial completion by species, trial duration, condition, and trial block bin. Point ranges indicate group means and 95% confidence intervals. Bins comprised 4 blocks (96 trials). Note that monkeys completed 10 bins of each condition, either the signalled condition first (bins 1–10) and then the unsignalled second (bins 11–20), or the unsignalled condition first (bins 1–10) and the signalled second (bins 11–20). Thus, each panel shows data from different monkeys in bins 1–10 and 11–20, depending on the order that they completed the conditions.

Across conditions and species, we also found changes in trial completion over time suggestive of learning (Fig. 3; for pairwise contrasts of the size of the coefficients, see Supplementary Table S4). Specifically, as testing progressed, monkeys became more likely to opt out of 5-s and 7-s trials (*b* ± *SE* 5 s: − 0.05 ± 0.004, 7 s: − 0.06 ± 0.004, *p*s < 0.001) but not 1-s trials (*b* ± *SE* = − 0.003 ± 0.004, *p* = 0.460). This effect was more pronounced in the signalled than in the unsignalled condition (*b* ± *SE* = − 0.04 ± 0.009, *p* < 0.001).

### Effect of time already spent tracking

**Figure 4:**
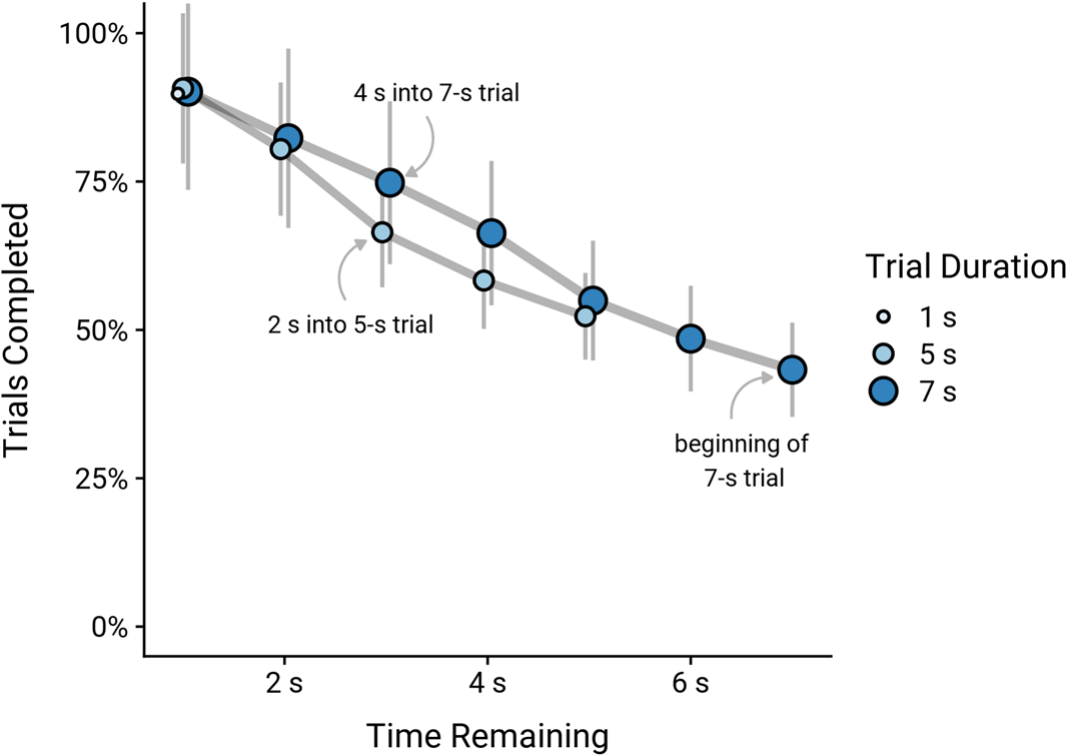
Trial completion by trial duration and time remaining. Point ranges indicate group means and 95% confidence intervals. Time remaining refers to durations of *at most* X seconds, e.g., if a monkey had tracked the target for 3.2 s, the sunk cost (time already spent tracking) was considered to be 3 s and the time remaining either 2 s (in a 5-s trial) or 4 s (in a 7-s trial).

We found a significant trial duration × time remaining interaction effect on monkeys’ likelihood to track the target for the required duration (Fig. 4), χ^2^(1) = 95.92, *p* < 0.001. Monkeys became more likely to complete the trial when less time was remaining, i.e., the longer they had already tracked the target. However, this effect was stronger in 5-s than in the 7-s trials (*b* ± *SE* 5 s: − 0.48 ± 0.01 vs. 7 s: − 0.37 ± 0.01, *p* < 0.001, pairwise contrast of the size of the coefficients: *b* ± *SE* = − 0.12 ± 0.01, *p* < 0.001). That is, in 7-s trials, in which sunk costs were by definition higher than in 5-s trials if the same time was remaining, the likelihood to complete the trial was less affected by the time remaining. In other words, for a given remaining time, monkeys were also more likely to finish tracking the target if they had already tracked it for longer.

## Discussion

Capuchin and rhesus monkeys showed pronounced sunk cost effects in this study. Instead of opting out and forfeiting their initial small investment, both species persisted 5–7 times longer than was optimal and were especially likely to complete a trial the longer they had already persisted. We found that rhesus macaques were more susceptible to sunk costs than capuchin monkeys and that both species showed more pronounced sunk cost effects when changes in trial duration were not signalled. These findings suggest that uncertainty plays a part in the emergence of this bias and highlight that qualitatively similar responses can still vary in magnitude both across and within species.

To our knowledge, this is the first demonstration of the sunk cost effect in nonhuman primates. Together with previous evidence of the effect in more distantly related species like pigeons, rats, and mice^8,18–21,23,24^, our results suggest that this bias can emerge as a result of evolved decision-making strategies that are widely shared across animal taxa and do not require human-unique processes, such as those underlying human language, formal economic markets, or self-rationalization. In particular, our finding that the sunk cost effect was more pronounced when changes in tracking duration were unsignalled indicates that the effect may arise because continued investment helps animals resolve uncertainty about future expected costs and benefits. Indeed, in pigeons, the sunk cost effect disappears completely when information about changes in the expected work requirement is provided^8^. However, in capuchin and rhesus monkeys, lack of information about when exactly it becomes optimal to opt out does not explain everything, as both species still showed pronounced sunk cost effects in the signalled condition.

One possible explanation is that primates overly rely on heuristics that use their past investment as a proxy for future costs and benefits or adjust how much they value an expected outcome based on their past investment. Heuristics are computationally simple rules of thumb that are likely to evolve if they work well in most situations that animals encounter, but they can sometimes lead to consistently suboptimal choices^39^. The sunk cost effect may arise as a result of such heuristics, especially when the cost is low compared to the optimal response (see Ref.^36^). Indeed, pigeons^8,18^, rats^19^, and humans in a similar paradigm^40^ are less susceptible to sunk costs when persisting to trial completion requires much more work than opting out to begin a new, likely shorter trial. These results suggest that the sunk cost effect only arises when it is “mildly” suboptimal to show it. Unlike pigeons and rats, capuchin and rhesus monkeys show this behaviour even when changes in expected effort are signalled, suggesting that they may be more reliant on heuristics. Nonetheless, we would expect these monkeys to stop persisting if it required even longer trial times (e.g., 15 or 30 s). The ratio of effort at which different species do so may provide a useful measure for comparison. Future work should compare other animals’, including humans’ responses to sunk costs in signalled versus unsignalled versions of the paradigm^8,40^ to assess this possibility empirically and assess to what extent this pattern may generalize.

This sensitivity in response to different task contingencies raises the question of how much animals understand about them and how they came to do so. Of course, they cannot be told the rules of the task ahead of time and need to learn over time through trial and error. Indeed, we found that monkeys’ responses in the test condition changed over time. They initially completed trials of all durations at high levels, as they did in training, but opted out of more 5- and 7-s trials (but not 1-s trials) as testing progressed. That is, their responses became more optimal over time. One possibility is that monkeys who easily learned to track the target (as evidenced by fewer trials before they met the training criteria) simply continued tracking the target in the test condition, too. For example, rhesus monkeys met the training requirement sooner and also completed test trials (showing a stronger sunk cost effect) at higher rates than capuchins. However, we found no statistical effect of training trials required on trial completion, nor can this explanation account for why they did not continue tracking trials of all durations at high levels or for the differences in signalling conditions. Alternatively, fast learners might be expected to also learn the contingencies of suboptimal versus optimal opting out, but this would not explain why monkeys of both species plateaued to complete at least 25% of 5- or 7-s trials at all. Thus, although learning certainly occurred, monkeys’ sunk cost effects in this study did not seem to arise from differences in learning.

Nonetheless, rhesus macaques showed a stronger sunk cost effect than capuchin monkeys in this study. In the unsignalled condition, all rhesus completed more 5- and 7-s trials than any of the capuchin monkeys, and they responded less to transitions in trial duration being signalled. We believe that differences in training performance underlie this difference in susceptibility to sunk costs in this task. However, such differences in training performance could also arise due to factors such as individual differences or differences in testing history and housing rather than inherent species differences. That rhesus monkeys reached the training criteria in many fewer trials than capuchins suggests that completing the task was generally easier for them to do. Therefore, the additional effort of tracking to trial completion may have presented even less of a cost to rhesus than to capuchin monkeys, favouring the sunk cost effect in this situation.

This possibility fits evidence from prior research that nonhuman primates may be more likely than humans to take a more optimal shortcut because the familiar, trained strategy is harder for them to learn and execute than it is for humans^35,41^. If so, we would expect humans in a comparable task to show even stronger sunk cost effects and would expect that the benefit from opting out compared to persisting would need to be larger than for rhesus macaques (whose in turn should be larger than for capuchins) in order for them to consistently opt out. Future comparative research should also extend this work to contexts other than continued motor action (for example, Ref.^24^) to investigate how general these effects are.

In this study, we report the first evidence for sunk cost effects in primates other than humans. We found that monkeys were less susceptible to the bias when transitions in expected additional work were signalled, indicating that animals may suboptimally persist, in part, because doing so resolves uncertainty about future outcomes. However, sunk cost effects emerged even when this uncertainty was removed and after continued experience with the task contingencies. We suggest that the sunk cost effect, a hallmark of human economic decision-making, may arise from evolutionarily ancient mechanisms that function to balance the costs and benefits for a given species’ cognitive abilities and environment.

## Supplementary information

Supplementary Video 1.

Supplementary Information 1.

## Data Availability

The data generated and analysed during this study are publicly available at the Harvard Dataverse (https://doi.org/10.7910/DVN/0YNZ0Q).
