# Supplementary figures and images for: Capuchin and rhesus monkeys show sunk cost effects in a psychomotor task
Source: Sci Rep. 2020 Nov 23;10:20396. doi: 10.1038/s41598-020-77301-w (PMC7683735; doi:10.1038/s41598-020-77301-w)

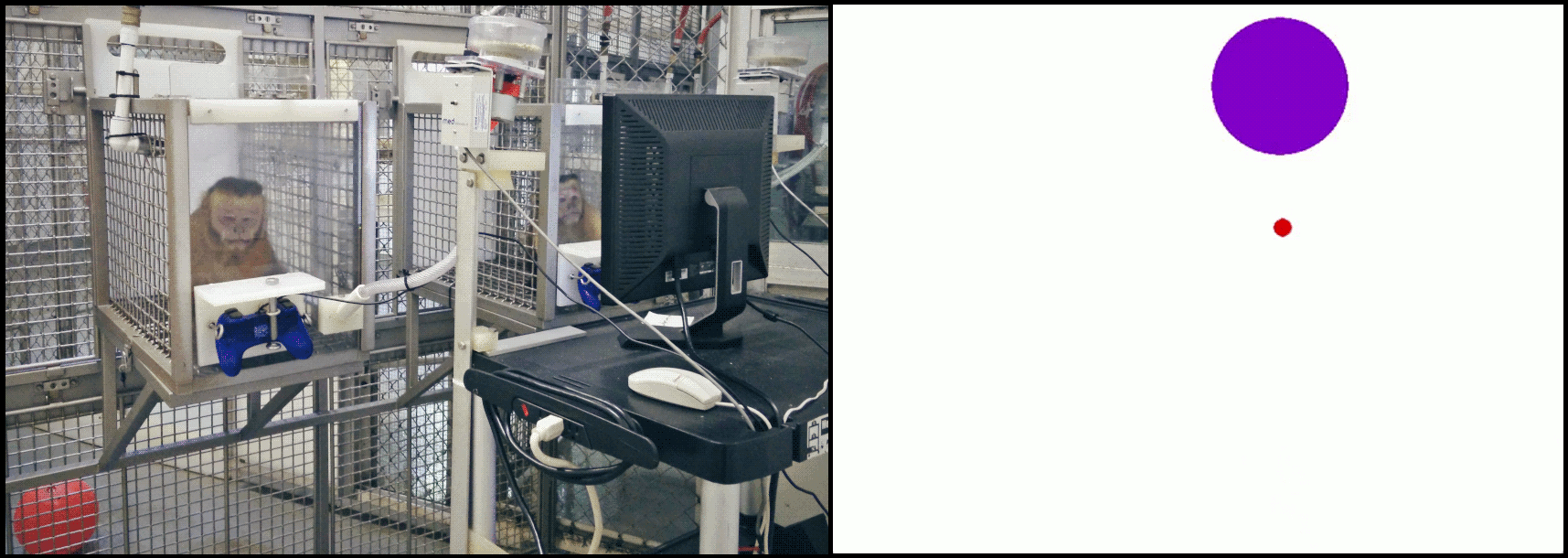

Supplement: Supplementary file 1 — Supplementary Video 1. [file 41598_2020_77301_MOESM1_ESM.gif]
