# Supplementary material for: Capuchin and rhesus monkeys show sunk cost effects in a psychomotor task
Source: Sci Rep. 2020 Nov 23;10:20396. doi: 10.1038/s41598-020-77301-w (PMC7683735; doi:10.1038/s41598-020-77301-w)
Supplement: Supplementary file 2 — Supplementary Information 1. [file 41598_2020_77301_MOESM2_ESM.pdf]

SUPPLEMENTARY INFORMATION

Capuchin and rhesus monkeys show sunk cost effects in a psychomotor task

Julia Watzek*[1], Sarah F. Brosnan[1,2]

[1]Department of Psychology, Language Research Center, Georgia State University, Atlanta, GA, USA
[2]Department of Philosophy, Neuroscience Institute, Center for Behavioral Neuroscience, Georgia State University, Atlanta, GA, USA

Model Specification

We specified the model testing the effects of trial duration and signalling condition on trial completion (Model 1) using the following model formula:

```
glmer(prop_earned_reward ~ (1|PID)
          + trial_duration * condition * species
          + trial_duration * condition * block_bin
          + z_training_duration,
      family = 'binomial',
      weights = num_trials,
      data = df)
```

After dropping the trial duration × condition × block bin term, the reduced model (keeping all two-way interactions) was specified using the following model formula:

```
glmer(prop_earned_reward ~ (1|PID)
          + trial_duration * condition * species
          + trial_duration * block_bin
          + condition * block_bin
          + z_training_duration,
      family = 'binomial',
      weights = num_trials,
      data = df)
```

We specified the model testing the effect of time already spent tracking on trial completion (Model 2) using the following model formula:

```
glmer(prop_earned_reward ~ (1|PID)
          + trial_duration * condition * species
          + trial_duration * remaining,
      family = 'binomial',
      weights = num_trials,
      data = df)
```

Supplementary Figure Caption

[The GIF file was uploaded separately, alongside the manuscript.]

Figure S1. Trial setup (left) and animated trial progression (right). Example trial shows screen capture of a 7-second trial in the signalled condition, in which the target was successfully tracked to trial completion.

Supplementary Tables

Table S1. Likelihood ratio tests for Model 1.
Terms significant at p < .05 are highlighted in grey.

Variable	χ^2	*df*	*p*
Trial duration × Condition × Species	17.79	2	< .001
Trial duration × Condition × Block bin	3.58	2	.167
Training duration	0.28	1	.597

Table S2. Likelihood ratio tests for the reduced Model 1.
Terms significant at p < .05 are highlighted in grey.

Variable	χ^2	*df*	*p*
Trial duration × Condition × Species	17.22	2	< .001
Trial duration × Block bin	103.07	2	< .001
Condition × Block bin	15.14	1	< .001

Table S3. Pairwise contrasts of estimated marginal means for Model 1: trial duration × condition × species. Contrasts at different levels of aggregation are computed across levels of the other factors; e.g., trial duration by species is computed across levels of condition (signalled & unsignalled). *OR* = Odds ratio. Terms significant at p < .05 are highlighted in grey.

**Trial duration**		*OR*	*SE*	*z*	*p*
	1 vs. 5 s	6.88	0.26	50.43	< .001
	1 vs. 7 s	10.25	0.39	60.97	< .001
	5 vs. 7 s	1.49	0.05	11.03	< .001
**Trial duration by Species**		*OR*	*SE*	*z*	*p*
Capuchin					
	1 vs. 5 s	11.45	0.45	61.85	< .001
	1 vs. 7 s	19.04	0.78	71.58	< .001
	5 vs. 7 s	1.66	0.07	12.86	< .001
Rhesus					
	1 vs. 5 s	4.13	0.27	21.64	< .001
	1 vs. 7 s	5.52	0.36	26.54	< .001
	5 vs. 7 s	1.34	0.08	4.78	< .001
**Trial duration by Species & Condition**		*OR*	*SE*	*z*	*p*
Capuchin, 1 s	signalled vs. unsignalled	1.03	0.06	0.50	.619
Capuchin, 5 s	signalled vs. unsignalled	0.42	0.02	-16.03	< .001
Capuchin, 7 s	signalled vs. unsignalled	0.35	0.02	-18.11	< .001
Rhesus, 1 s	signalled vs. unsignalled	1.10	0.11	0.94	.349
Rhesus, 5 s	signalled vs. unsignalled	0.79	0.07	-2.63	.009
Rhesus, 7 s	signalled vs. unsignalled	0.67	0.06	-4.71	< .001

Table S4. Pairwise contrasts of estimated marginal block bin trends for Model 1: trial duration × block bin and condition × block bin. Terms significant at p < .05 are highlighted in grey.

	b	*SE*	*z*	*p*
**Trial duration**				
1 - 5 s	0.05	0.01	8.43	< .001
1 - 7 s	0.06	0.01	9.25	< .001
5 - 7 s	0.01	0.01	1.01	.572
**Condition**				
signalled - unsignalled	-0.04	0.01	-3.89	< .001
